# Does rapid urbanization aggravate health disparities? Reflections on the epidemiological transition in Pune, India

**DOI:** 10.3402/gha.v7.23447

**Published:** 2014-09-09

**Authors:** Mareike Kroll, Erach Bharucha, Frauke Kraas

**Affiliations:** 1 Institute of Geography, University of Cologne, Cologne, Germany; 2 Institute of Environment Education and Research, Bharati Vidyapeeth Deemed University, Pune, India

**Keywords:** epidemiological transition, health disparities, India, low- and middle-income countries, urban health, urbanization

## Abstract

**Background:**

Rapid urbanization in low- and middle-income countries reinforces risk and epidemiological transition in urban societies, which are characterized by high socioeconomic gradients. Limited availability of disaggregated morbidity data in these settings impedes research on epidemiological profiles of different population subgroups.

**Objective:**

The study aimed to analyze the epidemiological transition in the emerging megacity of Pune with respect to changing morbidity and mortality patterns, also taking into consideration health disparities among different socioeconomic groups.

**Design:**

A mixed-methods approach was used, comprising secondary analysis of mortality data, a survey among 900 households in six neighborhoods with different socioeconomic profiles, 46 in-depth interviews with laypeople, and expert interviews with 37 health care providers and 22 other health care workers.

**Results:**

The mortality data account for an epidemiological transition with an increasing number of deaths due to non-communicable diseases (NCDs) in Pune. The share of deaths due to infectious and parasitic diseases remained nearly constant, though the cause of deaths changed considerably within this group. The survey data and expert interviews indicated a slightly higher prevalence of diabetes and hypertension among higher socioeconomic groups, but a higher incidence and more frequent complications and comorbidities in lower socioeconomic groups. Although the self-reported morbidity for malaria, gastroenteritis, and tuberculosis did not show a socioeconomic pattern, experts estimated the prevalence in lower socioeconomic groups to be higher, though all groups in Pune would be affected.

**Conclusions:**

The rising burden of NCDs among all socioeconomic groups and the concurrent persistence of communicable diseases pose a major challenge for public health. Improvement of urban health requires a stronger focus on health promotion and disease prevention for all socioeconomic groups with a holistic understanding of urban health. In order to derive evidence-based solutions and interventions, routine surveillance data become indispensable.

Rapid urbanization, which is altering the physical and social habitat of cities, is considered as one of the most important global health issues of the twenty-first century (1). Though not specifically addressing urban areas, the epidemiological transition theory, developed by Omran (2, 3), was one of the first models to describe and explain global trends in the dynamic relationship between demographic changes and epidemiological phenomena. The model is based on the assumption that major causes of death change unilinearly from communicable to non-communicable diseases (NCDs) within a society over time due to socioeconomic and medical progress (4). Based on Omran's concept (Fig. 1), Caldwell (5) developed the health transition concept taking into consideration different social, economic, and environmental development processes of societies, which can lead to a state of sustainable health, of improved medical technology, or of emerging and re-emerging infectious diseases (6).

**Fig. 1:**
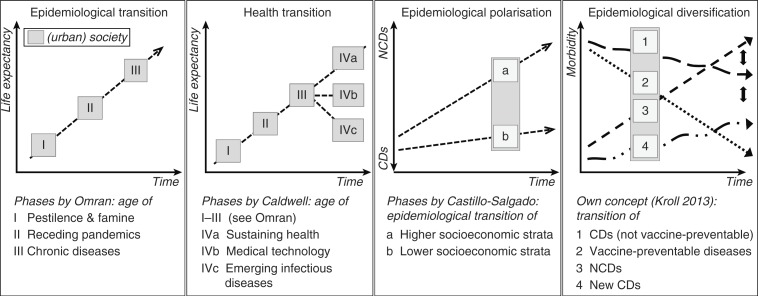
Different models of epidemiological transition. The first three graphs depict the epidemiological transition concepts of Omran (2, 3), Caldwell (5, 6), and Castillo-Salgado (11). The model of epidemiological diversification has been developed based on own findings in Pune in the context of rapid urbanization and steep socioeconomic disparities within the urban society. Source: Modified from Kroll (31).

Both theories are inadequate for application to rapidly growing cities in low- and middle-income countries, because these settings are characterized by increasing socioeconomic disparities and spatial fragmentation (7, 8). Health disparities (9) between different population subgroups coexisting in close proximity in cities (1) necessitate the inclusion of social and spatial components into the epidemiological transition concept (10). Castillo-Salgado addressed the increasing societal fragmentation in his concept of epidemiological polarization, which is characterized by ‘(…) a prolonged coexistence of two mortality patterns, one typical of the developed societies (chronic and degenerative), and the other of poor societal living conditions (infectious and parasitic) combined with high mortality from accidents and violence’ (11, p. 2). Whereas Castillo-Salgado referred to processes in North and South America, Shukla (12) identified similar patterns for the Indian urban society.

NCDs contributed to an estimated 53% of deaths in India in 2008 (13) and 44% of disability-adjusted life-years (DALYs) lost in 2005 (14) and are expected to increase significantly in the future (15). However, empirical evidence for the epidemiological transition of urban societies, and especially specific population subgroups, is limited because of a lack of reliable morbidity and mortality data (16, 17). Radkar et al., for example, described the epidemiological transition in urban Maharashtra using mortality data from 1981 to 2006. The analysis shows a slight decrease of communicable diseases, whereas diseases of the circulatory system, neoplasms, and injuries increased significantly (18). Comprehensive studies examining the epidemiological transition of specific urban population subgroups in India are non-existent, according to the authors’ knowledge, because of two factors. First, routine surveillance systems almost entirely address communicable diseases, and population surveys such as the National Family Health Survey are predominantly aggregated and do not reflect socioeconomic disparities in cities (19, 20). Second, intra-urban differences in health status have been largely neglected in India over recent decades, because the urban health care infrastructure was wrongly deemed to be adequate, with the focus being directed onto rural areas (21, 16). Therefore, only a small number of studies have thus far addressed current urban health disparities in India (7, 22).

Against this background, this study aimed to analyze the changing disease burden and health disparities between different socioeconomic groups in the emerging megacity of Pune.

## Methods

Pune, located in the state of Maharashtra, serves as an example of a rapidly urbanizing city undergoing huge transformation processes since the beginning of the 1990s due to economic and population growth. The population doubled between 1991 and 2011 to 3.1 million (23). Pune city is part of the Pune Urban Agglomeration, which is India's eighth largest metropolitan area with 5.1 million inhabitants (24).

Because health data availability for Pune is limited, a mixed-method approach was applied. While the description of epidemiological changes was mainly based on secondary mortality data, primary morbidity data were collected for the analysis of health disparities because of a lack of disaggregated data for different socioeconomic groups.

### Mortality data based on death registers

Mortality data were obtained from the Medical Certification of Causes of Death Scheme for the city of Pune during 1991 and 2006. Because only the 2006 data were standardized according to the International Classification of Diseases (ICD-10), the 1991 data were also grouped accordingly to facilitate comparability. All deaths without a registered cause in 1991 (32.6%) were excluded from the calculation.

### Morbidity data based on household survey

A household survey was conducted in two consecutive fieldwork phases in 2008 and 2009 in six neighborhoods in Pune to assess the disease burdens of different socioeconomic strata. The areas were selected according to spatial and social parameters: one registered slum (slum A) and one traditional middle class neighborhood (middle class A) in the old city center; one unregistered slum (slum B) and an upper middle class neighborhood (upper middle class B) in the former British cantonment; and an upper middle class neighborhood (upper middle class C) and a registered slum (slum C) at the urban fringe.

The survey was conducted in 900 randomly selected households. In each area, the total number of all households per street was counted and 150 households were systematically selected, choosing every n^th^ household from a starting landmark through a random route procedure (25). If no adult permanent household member was present, no appointment for a subsequent visit given or the interview denied, the next household was selected.

The objective of the survey was to assess the self-reported morbidity of all household members (in total 3,857 people) for the period of one year and the impact of major health determinants (e.g. diet, smoking). Regarding the health outcome, we focused on three lifestyle-related NCDs with a high incidence (diabetes, cardiovascular, and chronic respiratory diseases), and three communicable diseases linked significantly to the urban environment (tuberculosis, gastrointestinal, and vector-borne diseases). Survey data were analyzed applying descriptive methods. The prevalence rates of self-reported morbidities were age-standardized because of different demographic profiles of the areas.

### Qualitative interviews

Forty-six survey participants were invited for in-depth interviews to assess their insights on changes of various health determinants (e.g. income or dietary changes) during the past 10 years. The interviewees were purposively selected to represent households with average and high disease burdens. Because of considerable variations in the health knowledge and disease perception of laypeople, semi-standardized expert interviews were also conducted with 37 health care providers on the changing disease burdens in different socioeconomic groups as observed in their daily work over the past 10 years. The experts included 20 general practitioners, who were the most frequently designated family physicians identified from the survey, and 17 medical specialists from three major private and one major public hospital in Pune. Furthermore, 15 interviews were conducted in the two registered slum areas with staff from anganwadis (government-funded mother and child care centers) and seven interviews were conducted with medical experts from NGOs about past and current health problems in slum areas. All interviews were recorded, transcribed, and the texts coded for interpretation using the software MAXQDA. Survey and interview data were validated through cross verification in order to overcome weaknesses and intrinsic biases of individual methods. Secondary morbidity data from research studies in Pune and other Indian cities and government reports were used for triangulation.

## Results

### Mortality transition

The mortality data for the city of Pune, according to the main chapters of ICD-10, showed an increase in NCDs from 1991 to 2006 (Table 1). Diseases of the circulatory system increased from 20.8 to 25.8%. Whereas the cause of death was not further specified for the data from 1991, acute myocardial infarction (7.1% of all deaths) and cerebrovascular diseases (4.2% of all deaths) were the leading causes of death in this group in 2006. Chapters 1 and 4, which also predominantly comprise NCDs, also had a growing share of the total mortality, with diabetes being the major cause of death in the letter chapter. Within the group of respiratory diseases, which went up from 6.6 to 9.9%, deaths due to infectious respiratory diseases decreased (e.g. pneumonia from 87 to 79%), whereas the percentage of chronic respiratory diseases rose from 13 to 21.3%.

**Table 1:** Major causes of death for Pune according to the chapters of ICD-10 1991 and 2006

ICD-10 codes	1991 (%)	2006 (%)
I Certain infectious and parasitic diseases	12.3	12.6
II Neoplasms	3.0	7.0
III Diseases of the blood and blood-forming organs and certain disorders involving the immune mechanism	2.2	1.1
IV Endocrine, nutritional, and metabolic diseases	1.8	4.1
VI Diseases of the nervous system	1.7	2.8
IX Diseases of the circulatory system	20.8	25.8
X Diseases of the respiratory system	6.6	9.9
XI Diseases of the digestive system	2.2	4.3
XIV Diseases of the genitourinary system	–	3.0
XVI Certain conditions originating in the perinatal period	6.7	2.4
XVIII Symptoms, signs, and abnormal clinical and laboratory findings, not elsewhere classified	27.9	13.8
XIX Injury, poisoning, and certain other consequences of external causes	14.7	11.9

Source: Medical Certification of Causes of Death Scheme, Vital Statistics Office, Pune.

Infectious and parasitic diseases remained roughly on the same level, with a minor increase from 12.3 to 12.6%. Within this chapter, the cause of death changed considerably, with 66% of all deaths in 1991 being caused by gastrointestinal diseases, followed by tuberculosis (29%), tetanus and rabies (2% each), measles (0.9%), and leprosy (0.2%). In 2006, the majority of deaths in chapter 1 were certified as tuberculosis (40%), followed by septicemia (30%) and HIV/AIDS (19%). The mortality from gastrointestinal diseases (2.2%) and vaccine-preventable diseases such as tetanus (0.3%), measles (<0.1%), and rabies (0%) decreased, whereas the mortality from malaria and dengue slightly increased from 0 to 1.3 and 0.6%, respectively. However, the percentage of deaths classified as symptoms, signs, and abnormal clinical findings, which is high, but decreased from 27.8 to 13.8%, suggests a distortion of the mortality data because of insufficient investigation of deaths. Despite this drawback, the mortality data account for an epidemiological transition with an increasing number of deaths from NCDs, whereas within the group of communicable diseases, the causes of deaths have changed considerably. Yet, the high socioeconomic fragmentation of the population in Pune might imply that epidemiological changes do not occur unilinearly.

### Health disparities and morbidity

The self-reported prevalence of diabetes in the population above 20 years of age (Table 2) shows a gradient from 7.8 to 7.3% in the two upper middle class areas to 1.8% in slum B. The self-reported morbidity of hypertension, a common comorbid condition in diabetes, exhibits a similar gradient in the same age group ranging from 8.9% in upper middle class C to 3.0% in slum B. A triangulation with secondary data (16, 26–28) and expert interviews suggests that the self-reported morbidity is likely to be underreported for diabetes and hypertension, especially in the slum areas. General practitioners, cardiologists, and diabetologists in Pune confirmed the increasing prevalence of diabetes and hypertension in all socioeconomic groups.

There was a time when diabetes, hypertension – you really had to look for it in the slum areas. It has increased, but not as much as it has increased in the upper class. (General practitioner, area C)

**Table 2:** Self-reported age-standardized prevalence rates for selected diseases in six areas in Pune

	Upper middle class B	Upper middle class C	Middle class A	Slum C	Slum A	Slum B
Diabetes (>20 years) (%)	7.3 (*n=*463)	7.8 (*n=*432)	5.4 (*n=*498)	4.4 (*n=*494)	4.4 (*n=*407)	1.8 (*n=*319)
Hypertension (>20 years) (%)	8.0 (*n=*463)	8.9 (*n=*432)	7.4 (*n=*498)	5.2 (*n=*494)	6.9 (*n=*407)	3.0 (*n=*319)
Asthma (%)	0.9 (*n=*577)	1.4 (*n=*576)	0.6 (*n=*660)	1.3 (*n=*799)	0.4 (*n=*707)	0 (*n=*538)
COPD (%)	0.1 (*n=*577)	0.5 (*n=*576)	0 (*n=*660)	0.5 (*n=*799)	0 (*n=*707)	0 (*n=*538)
Malaria (%)	0.1 (*n=*577)	0.8 (*n=*576)	0.3 (*n=*660)	0.2 (*n=*799)	0 (*n=*707)	0 (*n=*538)
Dengue (%)	0.2 (*n=*577)	0.3 (*n=*576)	0.2 (*n=*660)	0 (*n=*799)	0 (*n=*707)	0 (*n=*538)
Tuberculosis (15–59 years) (%)	0 (*n=*354)	0.2 (*n=*354)	0 (*n=*396)	0.3 (*n=*453)	0.2 (*n=*364)	0 (*n=*307)
Gastrointestinal diseases (%)	1.8 (*n=*577)	0.6 (*n=*576)	1.1 (*n=*550)	0.7 (*n=*799)	0.7 (*n=*707)	0 (*n=*538)

Source: own survey 2008–09, *n=*3.857.

The prevalence was generally estimated to be higher among the affluent strata because of a wider diffusion of modern risk factors such as overweight and stress. Overweight according to body mass index was more prevalent in the two upper middle class areas (24% of all respondents compared to 4.5% in the two registered slums). However, it was assumed that the diabetes gradient is likely to change in the next decades because of an observed higher incidence in lower socioeconomic strata. This trend was ascribed to increasing health awareness in the upper class and an increasing susceptibility of lower socioeconomic groups because of an increasing impact of modern risks such as unhealthy diet, less exercise, alcohol consumption, smoking, and stress. Psychological problems for example – though tabooed – are common due to the stressful living conditions. More than half of the survey respondents in the three slum areas reported anxiety with respect to their current living conditions, compared to 20 to 30% in the middle class areas.

It's mostly tension. I'm not able to work then. There's no proper toilet in this area, no electricity most of the time, so it's a problem. This causes my blood pressure to rise. (Women, registered slum)

Furthermore, practitioners reported a higher incidence of comorbidities in diabetics of lower socioeconomic status. Besides common comorbid conditions such as ischemic, hypertensive, and renal diseases, non-healing wounds and also tuberculosis due to inadequate or non-existent disease management were mentioned. A diabetologist from a public hospital with predominantly patients of lower socioeconomic status stated:

Our patients come with the complications most of the time. (…) most of the diabetes damages like to the kidney or the eyes or to the heart are more or less permanent. (Diabetologist, public hospital)

The self-reported morbidity of asthma was the highest in upper middle class C (0.14%) and slum C (0.13%). No cases were reported in slum B, which is likely to be because of a lack of disease knowledge and under-diagnosis. Instead, many household members in slum B complained about frequent coughing (15.6%). Data from the National Family Health Survey 2005–06 for urban Maharashtra (29) showed an asthma prevalence of 1.5% among women and 1.9% among men, indicating an underreporting in all six areas. COPD was also the highest in upper middle class C (0.05%), followed by slum C (0.03%). General practitioners and chest physicians reported an increasing number of patients with chronic respiratory diseases in their daily practice in all socioeconomic strata. Whereas the increasing outdoor air pollution affects all strata, risk factors such as tobacco consumption (36.3% of all men above 20 years in the slum areas smoked daily compared to 17.9% in the middle class areas) and indoor air pollution due to substandard housing are more prevalent among slum dwellers. Furthermore, general practitioners and specialists reported that the disease would often be detected much later due to lack of disease knowledge and limited access to health care. Therefore, hospitalization and death would be more prevalent among the urban poor.

The malaria prevalence was the highest in slum B (3.7%), followed by upper middle class C (0.8%), and the lowest in slum A (0%). The data show high spatial variation but no socioeconomic pattern. A similar picture applies to dengue fever. Compared to the city-wide morbidity of malaria (0.07%) and dengue (0.002%) in 2009, the prevalence rates in the six areas were higher both for malaria (0.8%) and dengue (0.15%). Despite obligation to notify the Pune municipality, many malaria and dengue cases treated in the private health care sector are not reported. Medical experts accounted an increasing prevalence of both malaria and dengue in Pune. General practitioners in area B and C, for example, attributed the increasing number of cases to the high number of construction sites in these areas providing breeding grounds for mosquitoes throughout the year.

This area is a developing area, so most of all the construction activities are taken place, there is water logging (…). Now, what I found is that malaria has increased. (General practitioner, area C)

Although higher socioeconomic groups have more opportunities to protect themselves against mosquitoes, vector-borne diseases affect all strata with spatial and socioeconomic variations.

Although the mortality data for tuberculosis show a rise in the number of deaths in Pune, medical specialists in Pune generally reported a decreasing mortality of tuberculosis because of improved treatment options and higher detection rates. The self-reported morbidity of tuberculosis in the age group 15 to 65 does not show a clear pattern: the prevalence was the highest in slum C (0.34%), followed by upper middle class C (0.24%) and slum A (0.23%). In the other three areas, no cases were reported. Because of a social taboo surrounding tuberculosis, underreporting is likely in all areas. Anganwadi and NGO workers in slum C reported about several tuberculosis cases.

If there is one case of TB in one household also other members get affected because of the congestion. Because of the attitude, people don't want to get treated, they don't want to tell others about their disease. (Anganwadi worker, slum A)

The data of the National Family Health Survey for Mumbai 2005–06, for example, showed a prevalence of 0.69% in slum areas and 0.46% in non-slum areas (29). Medical practitioners in Pune still considered tuberculosis as a major problem, especially in slum areas because of high population densities and partly weak immune status due to poor living conditions and poor nutritional status. As for the latter, the consumption of vegetables and especially fruits was very low in the slum areas.

We sometimes eat fruits, in case somebody falls ill we eat fruits. I find it important to eat fruits but how could the poor ones afford it? (Woman, unregistered slum)

However, specialists in tertiary care hospitals also reported tuberculosis cases in higher socioeconomic strata mentioning exposure to pathogens at work and immunodeficiency due to high stress levels or chronic disease conditions, which increase the susceptibility, as the main reasons. But practitioners reported differences in the disease course with a higher number of drug and multi-drug resistances in lower socioeconomic strata due to non-compliance.

There are various reasons. One is socioeconomic status, they are not affording the treatment, or the patients are not aware, once they feel better they stop the treatment on their own. (Chest physician, private hospital)

The decreasing mortality from gastrointestinal diseases indicates a decreasing severity. The self-reported prevalence for gastrointestinal diseases does not show a clear trend: the highest prevalence was in slum B (2.2%) and upper middle class B (1.8%), and the lowest in upper middle class C (0.6%). All persons visited a doctor for treatment, indicating a certain severity of the disease. General practitioners considered gastrointestinal diseases as the major disease burden in their daily practice, together with infectious respiratory diseases. Although risk factors such as unhygienic conditions and contaminated drinking water pose a bigger problem in slum areas, practitioners and NGO experts also mentioned the huge improvements in living conditions in many registered slums over the last 10 years, resulting in a decreasing number and severity of gastrointestinal diseases.

Diarrhea is much less of a problem today than it used to be. (…) Even water supply has improved comparatively. (NGO expert)

This progress does not apply to unregistered slums, as proven by the survey, which usually lack adequate water supply. The changing life style, such as an increasing consumption of street and restaurant food, poses a risk for all socioeconomic strata.

### Epidemiological changes within socioeconomic groups

The longitudinal and cross-sectional data show a different progress of the epidemiological transition for distinct socioeconomic groups. The upper middle class is most likely to undergo the epidemiological transition as described by Omran:

So high blood pressure, diabetes, cardiac problems etc., all the gyms that you see in Pune city. (…) So this is one part of the population which is basically moving in the same direction as the West for example, in terms of their epidemiological profile. (NGO expert)

Nevertheless, this group is not completely safe from communicable diseases. Though the upper middle class has the necessary means to protect themselves or deal with infrastructural deficits, they are not completely protected from traditional risks such as contaminated water or pathogens in a highly fragmented urban society.

Among the traditional middle class, many health problems such as malnutrition or communicable diseases have been reduced within one generation due to improvements in the income situation, housing quality, and other factors. At the same time, cultural norms and social control prevent this group from some behavioral risk factors such as tobacco or alcohol abuse, which was least prevalent in this group. Therefore, the incidence of lifestyle-related diseases such as diabetes and hypertension is lower compared to the upper middle class.

And middle income groups is a balance of both actually although they are not very burdened with the infectious diseases (…). So the lifestyle disorders have now started streaming into the middle class as well. (Medical superintendent, private hospital)

In those registered slum areas, that received an adequate infrastructure in the last decade, the susceptibility to and severity of many communicable diseases decreases:

They have been provided with the basic amenities such as drinking water, waste disposal and health programs. So their health status is improving. This has a great impact. (General practitioner, area A)

At the same time, the incidence of NCDs rises because of an increasing impact of modern risks such as unhealthy diet, smoking, or stress. This is particularly problematic in view of the very limited financial capacity in this group to cover long-term medical treatment costs, which can push a household below poverty line. Therefore, people are, for example, at higher risk of uncontrolled diabetes, which increases the susceptibility to communicable diseases. Furthermore, results from the survey show that spending on tobacco or fast food was often at the expense of basic needs. Therefore, not only material but also behavioral factors play an important role for health protection among the urban poor.

This also holds true for the urban poor living in unregistered slum areas, which are exposed to most health risks due to lack of infrastructure and poor living conditions, but have the least capacity to sustain their health. Their susceptibility towards communicable diseases and accidents is the highest.

Malnutrition is a serious problem, their overall diets are inadequate and unhealthy, anemia is a serious problem, they have occupational problems, work related injuries, and they would have classical infectious diseases, high infant mortality, and they also start to experience some of the newer problems like HIV/AIDS. (NGO expert)

## Discussions

### Conceptual consideration of the epidemiological transition

Both secondary and primary data document a steady, unilinear rise of NCDs in Pune, especially diabetes, cardiovascular diseases, cancers, and chronic respiratory diseases, with slightly higher prevalence rates in higher socioeconomic strata. At the same time, vaccine-preventable diseases such as rabies or measles have been decreasing steadily. Other communicable diseases, which are not vaccine preventable (e.g. gastroenteritis) and can only be eliminated by improving the overall living conditions, still show a high morbidity with decreasing mortality. The burden of some communicable diseases such as dengue fever is growing in the course of urbanization. Malaria is again on the rise. Furthermore, new communicable diseases have emerged: HIV/AIDS prevalence has been steadily increasing over the last two decades and epidemics such as H1N1 with genetically evolved strains pose specific periodic risks. Although lower socioeconomic groups still have a higher susceptibility to communicable diseases, higher socioeconomic groups are also affected.

Moreover, epidemiological changes exceed quantitative changes in the disease burden and are also characterized by changes in the disease course. Examples include the declining severity of gastrointestinal diseases or the rising prevalence of diabetes and cardiovascular diseases in young people. Furthermore, interdependencies, or rather comorbidities, are increasingly occurring between the disease groups, for example, tuberculosis, HIV/AIDS, and diabetes. A growing incidence of multi-drug resistances in tuberculosis, malaria, HIV/AIDS, and leprosy pose unique challenges for the public health sector.

Looking at the epidemiological changes in Pune over the last decade, Castillo-Salgado's theory of epidemiological polarization does not apply. The epidemiological transition in Pune is characterized by different multilinear processes leading to increasing health disparities being characterized by many more interdependent factors than the quantitative disease burden alone. First of all, the exposure to health risk factors differs depending on the socioeconomic background. Overall, it was noted that some health determinants change in a linear fashion, but at a different pace according to the socioeconomic background (e.g. education, affordability of medical treatment). Other factors, especially environmental factors (e.g. air pollution) and some social factors (e.g. stress) increase for all groups at the same pace, although higher socioeconomic groups have more possibilities to minimize these risks. Furthermore, health knowledge and associated health-seeking behavior within the individual's scope influence the occurrence of a health event. These factors determine an unequal disease burden between socioeconomic groups. But even for the same acute or chronic condition, the disease course shows a socioeconomic gradient with a higher prevalence of complications or comorbidities in lower socioeconomic groups. This pattern is also linked to health care-seeking behavior: When visiting a health care facility, lower socioeconomic groups in particular tend to visit a less or non-qualified practitioner, and are therefore at greater risk of inadequate diagnosis and management (30). Furthermore, the risk of inadequate medication is rather high because of financial constraints (e.g. long-term treatment for diabetes is not affordable) or low locus of control and health knowledge (e.g. cost-free DOTS-therapy for tuberculosis is discontinued or medication is intermittent). Additionally, the treatment outcome is influenced by the disease behavior (e.g. dietary changes in case of diabetes).

Overall, socioeconomic subgroups in Pune show different epidemiological profiles which are not isolated, but rather interdependent on various levels, for example, through transmission routes of pathogens in a highly fragmented urban environment or structural inequities within the urban society influencing the exposure to health risks. The epidemiological transition does not proceed unilinearly, but rather exhibits different trends with linear, periodic, and recurrent elements (Fig. 1). Therefore, the terminology of epidemiological diversification appears more appropriate for rapidly urbanizing societies with profound socioeconomic disparities.

### Limitations of the study

The results of the study have to be interpreted carefully because of several methodological limitations. First of all, mortality data could only be obtained for specific years and not for a complete period. The data from 1991, in particular, are distorted because of the high number of deaths, which were not certified at all or certified as symptoms, signs, and abnormal clinical and laboratory findings. Furthermore, progress in medical diagnostics and improved access to health care restrict the comparability. The same bias applies to expert interviews on the changing disease burden in Pune. Medical specialists drew attention to the problem of increasing diagnostic opportunities leading to higher detection rates, that is, not necessarily indicating rising incidence rates. In addition, medical experts described changes in the disease burden of different socioeconomic groups based on experience in everyday practice, and results might therefore be subjectively distorted.

Furthermore, the analysis of health disparities through household survey data has several methodological limitations. First, residential areas are not socioeconomically homogeneous entities, and therefore status inconsistencies are always possible. Nevertheless, the differences between the six areas with respect to housing quality, infrastructure supply, and income levels are notable. Second, the selection of households by random walk in densely populated and fragmented areas without proper addresses has its limitations. Therefore, only households with an adult household member present were considered for the survey. Third, the comparability of the results from the household survey is limited because of the differences in health knowledge and disease perceptions of different socioeconomic groups, resulting in a different reporting behavior on health status and health risks. For example, lower socioeconomic groups tend to have poorer access to the health care system and less health knowledge (information bias) (19) and are prone to underreport or wrongly report certain health problems.

Despite these drawbacks, the mixed methods approach helped to an extent to overcome the lack of secondary disaggregated morbidity data and to draw conclusions on epidemiological changes of different urban population subgroups (32).

## Conclusions

The rapid urbanization process in Pune has an impact on urban health in various ways: high population densities, environmental and infrastructural degradation, unequal access to health care, and strong social stratification and fragmentation, as observable in many cities in low- and middle-income countries, jeopardize improvements in disease control and lead to an epidemiological diversification. In contrast to existing unilinear or bipolar approaches, the epidemiological transition in Pune is characterized by multilinear processes, which seem to aggravate health disparities among different socioeconomic groups.

Growing disparities and an increasing burden of NCDs in urban populations require a stronger focus on health promotion and disease prevention catering to different needs of population subgroups. Adequate disease management by strengthening the public health care sector is required especially for lower socioeconomic groups. Undetected and inadequately managed NCDs might interfere with disease control of chronic communicable diseases in the future. In order to derive appropriate solutions, routine disease surveillance systems are needed which should also capture spatial and socioeconomic data in order to develop adequate interventions for different risk groups to counteract widening health disparities.
